# Septin Remodeling During Mammalian Cytokinesis

**DOI:** 10.3389/fcell.2021.768309

**Published:** 2021-11-04

**Authors:** Giulia Russo, Michael Krauss

**Affiliations:** Leibniz-Forschungsinstitut für Molekulare Pharmakologie (FMP), Berlin, Germany

**Keywords:** septins, cytokinesis, actomyosin, microtubules, phosphoinositides

## Abstract

Cytokinesis mediates the final separation of a mother cell into two daughter cells. Septins are recruited to the cleavage furrow at an early stage. During cytokinetic progression the septin cytoskeleton is constantly rearranged, ultimately leading to a concentration of septins within the intercellular bridge (ICB), and to the formation of two rings adjacent to the midbody that aid ESCRT-dependent abscission. The molecular mechanisms underlying this behavior are poorly understood. Based on observations that septins can associate with actin, microtubules and associated motors, we review here established roles of septins in mammalian cytokinesis, and discuss, how septins may support cytokinetic progression by exerting their functions at particular sites. Finally, we discuss how this might be assisted by phosphoinositide-metabolizing enzymes.

## Introduction

**FIGURE 1:**
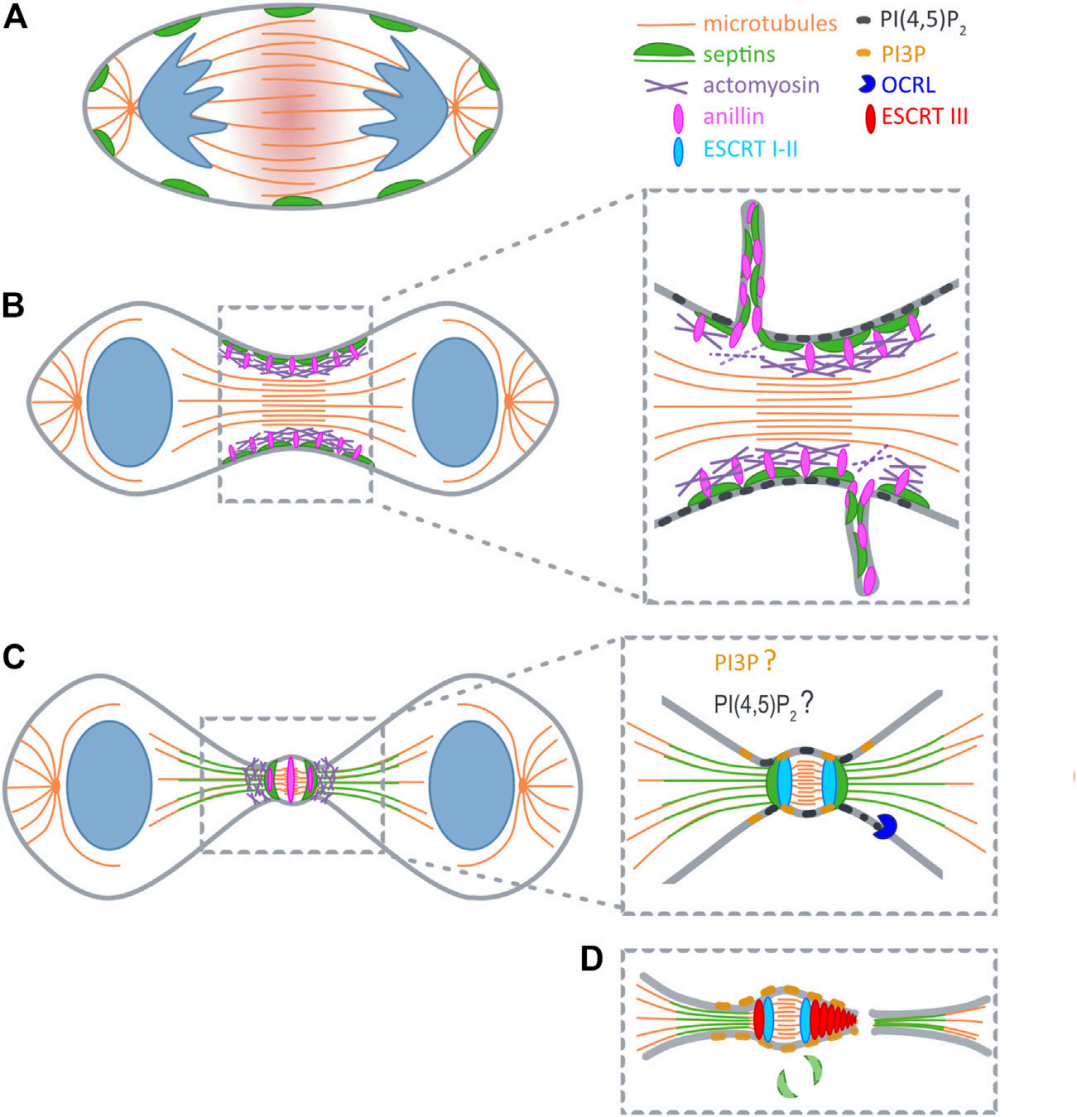
Septins during cytokinesis. **(A)** At anaphase RhoA is activated at the midzone (red shadow). **(B)** At early telophase septins (green) are recruited to furrow by anillin (pink), and during furrow constriction promote extrusion of plasma membrane tubules. **(C)** After ingression septins reorganize into a double ring and support the formation of secondary ingression sites by dictating the relocalization of anillin and actomyosin (purple). At the same time SEPT9 locally activates Tsg101 (light blue). Initial recruitment of ESCRTI to the midbody is not depicted for clarity reasons. **(D)** Recruitment of ESCRTIII (red) occurs concomitantly with the disappearance of septin double rings. PI(4,5)P_2_ (dark grey) builds up early at the cleavage furrow, and might also become concentrated at the midbody ring. PI(4,5)P_2_ is hydrolyzed upon arrival of OCRL (blue) at the ICB. Prior to abscission a pool of PI(3)P (yellow) is generated at the midbody that likely supports ESCRT assembly.

Cytokinesis starts when the centralspindlin complex activates Rho A at the equatorial plane of the cell cortex to initiate the formation of the contractile machinery (Glotzer, 2009; Zhang and Glotzer, 2015) (Figure 1A). GTP-loaded Rho A associates with anillin, a scaffolding protein that can roughly be divided into two functional halves (Carim et al., 2020): The N-terminus coordinates the recruitment of formins, actin, myosin II and Rho-dependent kinase to trigger actin polymerization and myosin II activation. Anillin thereby orchestrates the assembly of a stable contractile ring (Piekny and Maddox, 2010). Through its C-terminal domain anillin associates with Rho A, PI(4,5)P_2_ and septins and drives the translocation of septins to the newly forming cleavage furrow (Oegema et al., 2000; Field C. M. et al., 2005; Liu et al., 2012; Sun et al., 2015).

Surprisingly, though present already at early stages of cytokinesis septins are dispensable for initial furrow ingression. Recent studies instead demonstrate key roles during the maturation of the cytokinetic bridge, and in the final step of abscission (see below). Here, we summarize our current knowledge of septin behavior and functions at different stages of cytokinesis, and discuss mechanisms underlying their redistribution during cytokinetic progression.

### Septin Functions During Cleavage Furrow Initiation and Ingression

Several lines of evidence indicated that septins function already at early stages of cytokinesis. Septins associate with anillin, a master regulator of actomyosin ring assembly (Carim et al., 2020), through its C-terminal pleckstrin homology (PH) domain (Field C. M. et al., 2005; Liu et al., 2012). Septin recruitment to the forming cleavage furrow requires anillin, and might be reinforced by anillin-dependent F-actin crosslinking (Kinoshita et al., 1997; Kinoshita et al., 2002; Matsuda et al., 2020) (Figure 1B). Consistent with a role at the actomyosin ring Joo et al. demonstrated that SEPT2 directly interacts with myosin II, and scaffolds myosin II association with Citron kinase and Rock to support activation of this motor (Joo et al., 2007). Surprisingly, however, even when complex formation of septins with myosin II is inhibited, cells manage to assemble a contractile ring, and the cleavage furrow ingresses. Only as soon as the ICB is formed, the furrow becomes destabilized and retracts (Joo et al., 2007). Similar observations were made in HeLa cells expressing an anillin chimera, in which the C-terminal PI(4,5)P_2_- and septin-binding PH domain is replaced by a PH domain that retains affinity for (PI4,5)P_2_, but cannot associate with septins (Renshaw et al., 2014). Together, these studies indicate that septins are not required for the assembly of the contractile ring itself, but rather contribute to its shrinkage, or promote its tethering to the midzone.

In line with this interpretation live cell imaging in HeLa cells, as well as in *Drosophila* S2 cells revealed internalization of packages of anillin and a subset of associated cytokinetic proteins, including septins, during maturation of the midbody ring (El Amine et al., 2013; Renshaw et al., 2014) (Figure 1B). In both systems this phenomenon depends on anillin’s capability to associate with septins. Based on their findings in *Drosophila* Carim et al. suggested that an anilloseptin subnetwork supports the reduction of circumference of the contractile ring (Carim et al., 2020). According to their model closure of the contractile ring builds tension that disengages anillin from the actomyosin network to induce extrusion of anilloseptin-containing plasma membrane tubules.

### Septins Prime the Abscission Machinery at Late Stages of Cytokinesis

Superresolution imaging revealed that during elongation of the ICB anillin and septins form a collar-like structure, that gradually elongates through the assembly of an array of circular (or helical) filaments oriented parallel to the plane of cell division (Renshaw et al., 2014). Subsequently, anillin translocates to the midbody, and – dependent on its interaction with septins – to two flanking rings (Figure 1C). Septins, on the other hand, are excluded from the midbody, but colocalize with anillin at the flanking rings (Karasmanis et al., 2019). Thus, septins and anillin appear to switch roles at this stage, with septins dictating the localization of anillin. At the double ring two secondary actomyosin rings are formed, which serve to further constrict the bridge to a diameter of 100–300 nm (Wang et al., 2019). These constriction sites might explain why septins serve a barrier function to prevent premature loss of cytokinetic proteins from the ICB by diffusional spread (Estey et al., 2010).

One of the secondary ingression sites will eventually evolve into the site of abscission. Abscission relys on the ESCRT machinery (Mierzwa and Gerlich, 2014; Addi et al., 2018), the recruitment of which occurs sequentially. First, ALIX and TSG101 associate with the midbody through their association with CEP55 (Lee et al., 2008). Both proteins subsequently support the recruitment of the ESCRTIII component CHMP4B in two parallel acting pathways (Christ et al., 2016). CHMP4B then translocates to the secondary ingression sites, in a step that depends on anillin, anillin’s interaction with septins, and, consequently, also septins themselves (Renshaw et al., 2014) (Figures 1C,D).

A recent study by Karasmanis et al. (2019) provided more detailed mechanistic insight into the impact of SEPT9 on ESCRT rearrangements within the ICB. As expected, loss of SEPT9 does not affect the enrichment of ALIX and TSG101 at the midbody, but severely impairs the assembly of ESCRTIII into symmetric rings flanking the midbody. As the authors found SEPT9 to associate directly with Tsg101, they hypothesized that SEPT9 locally activates Tsg101 to facilitate complex formation with downstream ESCRTs and to coordinate their stepwise assembly. Indeed, in absence of SEPT9 ESCRTIII fails to reassemble into the characteristic cone-shaped structures that promote abscission. Intriguingly, both septins organized in rings, and ESCRT-III are comprised of arrays of regularly spaced, 10–20 nm wide filaments (Guizetti et al., 2011; Ong et al., 2014). These similarities in dimension and arrangement raise the possibility that septin rings act as a template for the formation of higher-order assemblies of ESCRT-III.

### Roles of Septin Association With Microtubules During Cytokinesis

Septins are found associated with microtubules in several cell types, to regulate their nucleation, organization, dynamics, posttranslational modification, the capture of their plus-ends at the cell cortex, and microtubule-dependent transport events (Spiliotis and Nakos, 2021). Here, we will summarize roles of microtubule-associated septins during chromosome alignment and segregation, and discuss how they may assist abscission at late stages of cytokinesis.

Chromosomes that fail to align at metaphase are captured by centromere-associated protein E (CENP-E), which has been shown to be a binding partner of SEPT7 (Zhu et al., 2008). The maintenance of CENP-E at kinetochore microtubules also requires the presence of SEPT2 that partially colocalizes with those microtubules in a network of short SEPT2-containing filaments found juxtaposed with kinetochores (Spiliotis et al., 2005). In line with a function of septins in chromosome segregation, loss of SEPT7, as of SEPT2, causes the mis-localization of CENP-E to the spindle poles, and results in defects in chromosome alignment at the metaphase plate (Spiliotis et al., 2005; Zhu et al., 2008).

Super-resolution imaging of MDCK cells, for instance, revealed that septins localize on microtubule portions distal to the midbody, but are excluded from the midbody itself (Karasmanis et al., 2019). The latter finding could reflect a competition by microtubule-associated proteins enriched at the midbody, including PRC1 and centralspindlin (Glotzer, 2009).

The molecular mechanisms underlying the redistribution of septins from the cortex to microtubules remain largely elusive, but some evidence suggests a regulatory role of Cdc42, and a modulatory function of posttranslational modifications. In interphase the maintenance of septins on actin stress fibers relies on active Cdc42 and Cdc42 effector proteins (Borgs). Cdc42 inactivation, or depletion of Borgs triggers septin redistribution onto microtubules (Salameh et al., 2021). During mitosis Cdc42 localizes to the central spindle at metaphase, to concentrate at the ICB at telophase (Oceguera-Yanez et al., 2005). Moreover, Cdc42 activity peaks at metaphase, and drops during telophase (Oceguera-Yanez et al., 2005). This suggests that local inactivation of Cdc42 at the ICB might release septins from cortical actin at late stages of cytokinesis. The subsequent translocation to microtubules might by driven by their preference for arrays of parallel, or stabilized microtubules (Spiliotis and Nakos, 2021). Further, the association of septins with microtubules appears to be modulated by posttranslational modifications, such as sumoylation (Ribet et al., 2017). Ribet et al. identified sumoylation sites in several human septins, and demonstrated that non-sumoylatable septin variants form aberrant bundles in interphase. During cytokinesis such bundles accumulate along the ICB during cytokinesis, where they probably align with microtubules, and cause late cytokinetic defects (Ribet et al., 2017).

The roles that septins play on bridging microtubules are gradually emerging. Given that SEPT9 promotes microtubule bundling *in vitro* (Bai et al., 2013), septins might facilitate bundling of microtubules at constriction sites, and – due to their interaction with PI(4,5)P2_2_ (see below) – aid microtubule attachment to the plasma membrane. Further, septins support microtubule severing that precedes abscission, as indicated by findings in fibroblasts derived from SEPT7 knockout mice (Menon et al., 2014). These fibroblasts display hyperacetylated microtubules, and show abscission defects. Importantly, the cytokinesis block can be bypassed by expression of stathmin, suggesting that SEPT7 facilitates the destabilization of microtubules within the ICB to prime them for severing.

Another level of regulation is suggested by the interplay between septins and kinesins (Spiliotis and Nakos, 2021). A recent study demonstrates that SEPT7 colocalizes and interacts with MKLP2/KIF20A (Qiu et al., 2020), a kinesin required for cleavage furrow stability at late stages of cytokinesis (Wu et al., 2019). Loss of SEPT7 depletes MKLP2 from the ICB in neural progenitor cells and leads to cell division defects (Qiu et al., 2020). It is, thus, not unlikely that septins similarly affect the localization and/or activity of other kinesin motors functioning at other stages of cytokinesis. For instance, septins could serve to locally concentrate the centralspindlin complex through associating with its motor component MKLP1/KIF23 (Mishima, 2016).

### Phosphoinositides Guide Cytokinetic Factors to the Plasma Membrane

Phosphoinositides (PIs) are differentially phosphorylated derivatives of the membrane phospholipid phosphatidylinositol (Krauss and Haucke, 2007). PI(4,5)P_2_ is prevalently present at the plasma membrane and builds up early at the newly forming cleavage furrow, leading to an about 4,5 fold increase in concentration at the ingressed cleavage furrow (Field S. J. et al., 2005) (Figure 1B). Conclusively, PI(4,5)P_2_ has been implicated in the recruitment of numerous cytokinetic factors, including centralspindlin (Lekomtsev et al., 2012), anillin (Liu et al., 2012; Sun et al., 2015) and septins (Zhang et al., 1999; Tanaka-Takiguchi et al., 2009).

To date, surprisingly little is known about the PI-kinases that generate pools of PI(4,5)P_2_ during cytokinesis, in particular of the ones that underlie the *de novo* formation of higher-ordered septin structures, or that promote their attachment at the cell cortex. In mammalian cells overexpressed PIPKIβ, a PI(4,5)P_2_-synthesizing enzyme, is found enriched at the cleavage furrow (Emoto et al., 2005), but it remains unclear, whether PIPK1β affects the distribution of cytokinetic factors.

At late stages of cytokinesis centralspindlin accumulates at the midbody (Hutterer et al., 2009). Its motor subunit MKLP1 forms complex with Arf6, and promotes its recruitment of this small GTPase to the midbody (Boman et al., 1999; Makyio et al., 2012). Of note, Arf6 associates with, and activates PIPKI enzymes (Honda et al., 1999; Krauss et al., 2003). This suggests that the MKLP1-Arf6 complex initiates the generation of a PI(4,5)P_2_ pool at the midbody to assist the concentration of PI(4,5)P_2_-binding, cytokinetic proteins at this locale.

Prior to abscission, PI(4,5)P_2_ at the ICB is hydrolyzed by Oculo-cerebro-renal syndrome of Lowe (OCRL) (Dambournet et al., 2011), a 5-phosphatase, that is recruited by endosomal Rab35, and delivered to the ICB to dephosphorylate PI(4,5)P_2_. Cells lacking expression of Rab35, or of OCRL, display defects in abscission, exhibit increased local levels of PI(4,5)P_2_ and accumulate F-actin at the ICB. By contrast, overexpression of dominant-negative Rab35 triggers accumulation of PI(4,5)P_2_, but also of septins on abnormal intracellular vacuoles formed before entry into mitosis (Kouranti et al., 2006). Taken together, these observations emphasize the key role of PI(4,5)P_2_ in guiding the subcellular distribution of septins, and suggest that the delivery of OCRL promotes the release of septins (and probably of anillin) from the cell cortex within the ICB. Likely, this also facilitates their association with microtubules (see above).

The final abscission reaction requires the stepwise assembly of the ESCRT machinery, some components of which depend on PI(3) for their association with at endosomal membranes (Yorikawa et al., 2005; Teo et al., 2006). PI(3)P also accumulates at the midbody during cytokinesis, where it recruits the centrosomal protein and PI(3)P-binding protein FYVE-CENT and its binding partner TTC19 (Sagona et al., 2010). TTC19 in turn interacts with CHMP4B, and accordingly, loss of TTC19 or of FYVE-CENT triggers late cytokinetic defects (Sagona et al., 2010). Based on their observations the authors of this study hypothesized that midbody-associated PI(3)P is delivered by fusion of endosomes with the plasma membrane at the ICB. Alternatively, and based on analogy (Posor et al., 2013), one might speculate that a plasma membrane-based PI conversion mechanism could involve the stepwise hydrolysis of PI(4,5)P_2_ into PI(4)P (through OCRL), towards the generation of PI(3)P, for instance through the synthesis of a PI(3,4)P_2_ intermediate.

### Future Directions

Although roles of septins during cytokinesis are firmly established, several aspects remain unclear. What are the molecular mechanisms underlying extrusion of anillin, septin and select cytokinetic proteins in tubules? Why do septins translocate to ICB microtubules, what is the functional relevance of this “hopping” behaviour, and why is it not observed in all cell types? How exactly is the translocation to microtubules modulated by posttranslational modifications, in particular by phosphorylation through mitotic kinases? Which motors, other than MKLP2/KIF20A, are retained at the ICB by septins? Which enzymes promote changes in phosphoinositide identity at the ICB? In particular, how are pools of PI(3)P and its derivatives generated, that appear at the ICB and support ESCRT-dependent abscission, and where exactly are these pools localized (Gulluni et al., 2017) (Figure 1D)? Given the dimensions of the ICB, answers to these questions will clearly rely on the application of super-resolution microscopy techniques.
